# Disseminated histoplasmosis mimicking hematologic malignancy in a patient with human immunodeficiency virus

**DOI:** 10.1002/jha2.389

**Published:** 2022-01-27

**Authors:** Michael E. Kallen, Ahmed Khalil, Peter Anthony DeRosa, Maria R. Baer

**Affiliations:** ^1^ Department of Pathology University of Maryland School of Medicine Baltimore Maryland USA; ^2^ Department of Medicine University of Maryland School of Medicine Baltimore Maryland USA; ^3^ University of Maryland Greenebaum Comprehensive Cancer Center Baltimore Maryland USA

**Keywords:** AIDS, Hematologic malignancy, Histoplasmosis, HIV


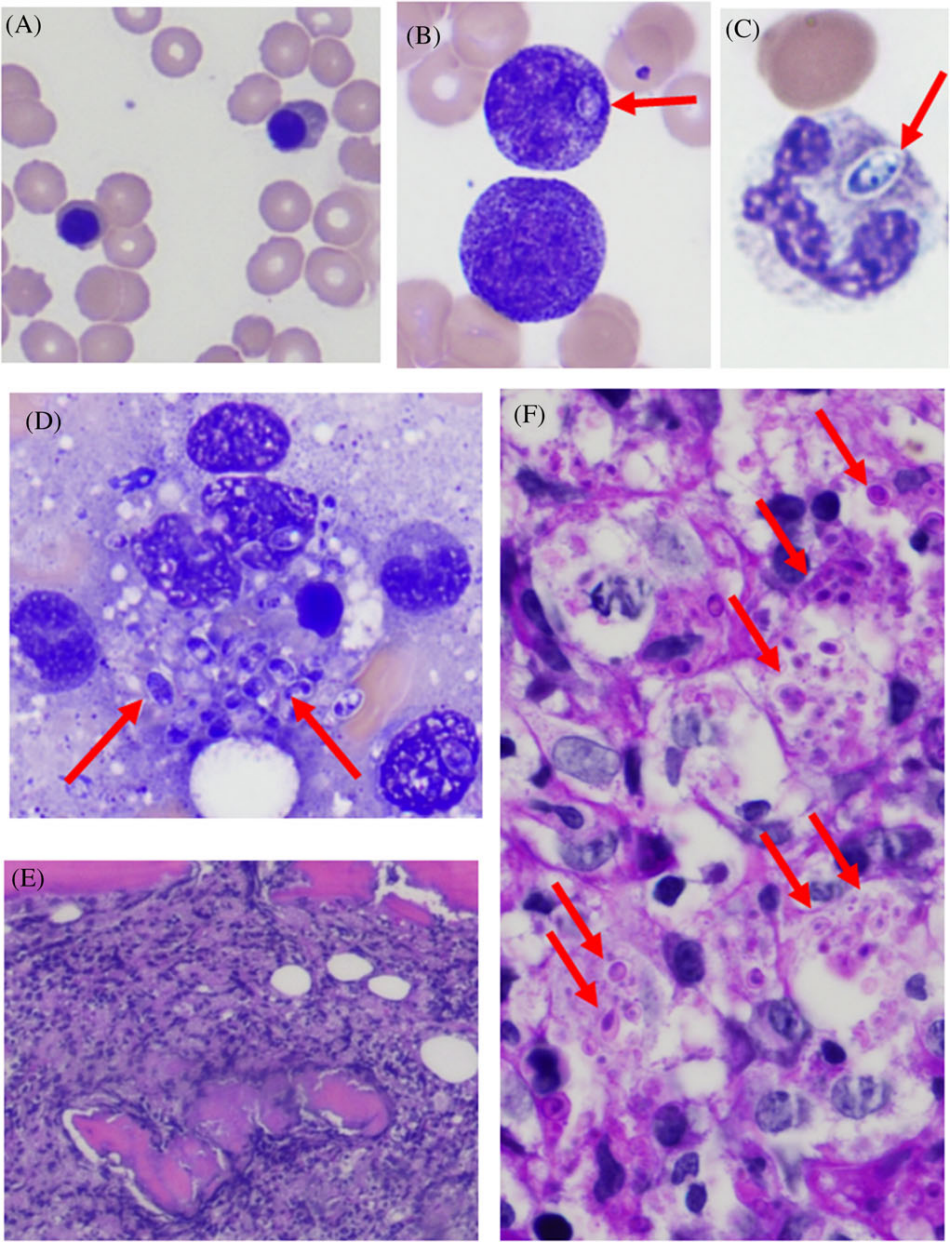


A 25‐year‐old recent immigrant from El Salvador with a known human immunodeficiency virus (HIV) infection was transferred to the University of Maryland Greenebaum Comprehensive Cancer Center with altered mental status, fever, epistaxis, gingival hemorrhage, melena, and rash. He was cachectic and acutely ill, with axillary and inguinal adenopathy and a palpable spleen tip. Hemoglobin was 66 g/L, white blood cell (WBC) count 6.6 × 10^9^/L with 38% segmented neutrophils, 10% bands, 11% metamyelocytes, 13% myelocytes, 6% blasts and 200 nucleated red blood cells per 100 WBC, and platelet count 64 × 10^9^/L. Creatinine was 472 μmol/L, lactate dehydrogenase 15,983 iu/L, and uric acid 702 μmol/L. HIV viral load was 82,040 copies/ml and absolute CD4 count was 16 cells/dl. Computerized tomography of the chest and abdomen showed extensive upper mediastinal and axillary adenopathy, bilateral pleural effusions, scattered retroperitoneal, mesenteric and inguinal adenopathy, splenomegaly, and ascites. The presumed diagnosis was a hematologic malignancy. However, the peripheral blood smear demonstrated not only leukoerythroblastic changes, including nucleated red blood cells (A) and myelocytes (B) but also intracytoplasmic yeast organisms in myelocytes (B) and neutrophils (C), and bone marrow aspirate (D) and biopsy (E, F) showed prominent histiocytosis with innumerable intracellular ovoid yeast forms. The patient was diagnosed with disseminated histoplasmosis involving blood, bone marrow, skin and cerebrospinal fluid, as well as mycobacterium avium complex and klebsiella septicemia, complicating uncontrolled HIV infection. He responded to antibiotics, including a 6‐week course of liposomal amphotericin B.

Uncontrolled HIV infection is rare in the setting of currently available highly active antiretroviral therapy, but does occur, and can be complicated by disseminated opportunistic infections, as well as by high‐grade lymphomas and other malignancies. Leukoerythroblastosis, defined by the presence of nucleated red blood cells and immature myeloid cells in the circulating blood, can be caused by malignant bone marrow infiltration, but also by disseminated infection. Our patient's bone marrow failure, leukoerythroblastosis, and extensive lymphadenopathy were caused by disseminated histoplasmosis, rather than by a hematologic malignancy.

## FUNDING INFORMATION

NIH‐NCI, Grant/award no: P30 CA134274.

## CONFLICT OF INTEREST

The authors declare that they have no conflict of interest.

